# Disentangling the Effects of Spinal Cord Injury and Related Neuropathic Pain on Supraspinal Neuroplasticity: A Systematic Review on Neuroimaging

**DOI:** 10.3389/fneur.2019.01413

**Published:** 2020-02-05

**Authors:** Vincent Huynh, Jan Rosner, Armin Curt, Spyros Kollias, Michèle Hubli, Lars Michels

**Affiliations:** ^1^Spinal Cord Injury Center, Balgrist University Hospital, University of Zurich, Zurich, Switzerland; ^2^Department of Neuroradiology, University Hospital Zurich, Zurich, Switzerland; ^3^Department of Neurology, Bern University Hospital (Inselspital), University of Bern, Bern, Switzerland; ^4^MR-Center, University Children's Hospital Zurich, Zurich, Switzerland

**Keywords:** neuroimaging, spinal cord injury, neuropathic pain, brain plasticity, systematic review

## Abstract

**Background:** Spinal cord injury (SCI) and its accompanying changes of brain structure and function have been widely studied and reviewed. Debilitating chronic neuropathic pain (NP) is reported in 53% of SCI patients, and brain changes have been shown to be involved with the presence of this secondary complication. However, there is yet a synthesis of current studies that investigated brain structure, resting connectivity, and metabolite changes that accompanies this condition.

**Methods:** In this review, a systematic search was performed using Medical Subject Headings heading search terms in PubMed and SCOPUS to gather the appropriate published studies. Neuroimaging studies that investigated supraspinal structural, resting-state connectivity, and metabolite changes in SCI subjects with NP were included. To this end, voxel-based morphometry, diffusion tensor imaging, resting-state functional MRI, magnetic resonance spectroscopy, and PET studies were summarized and reviewed. Further inclusion and exclusion criteria allowed delineation of appropriate studies that included SCI subgroups with and without NP.

**Results:** A total of 12 studies were eligible for qualitative synthesis. Overall, current studies that investigated NP-associated changes within the SCI cohort show primarily metabolite concentration alterations in sensory-pain processing regions, alongside bidirectional changes of brain structure. Moreover, in comparison to healthy controls, there remains limited evidence of structural and connectivity changes but a range of alterations in metabolite concentrations in SCI subjects with NP.

**Conclusions:** There is some evidence suggesting that the magnitude and presence of NP following SCI results in both adaptive and maladaptive structural plasticity of sensorimotor regions, alongside altered metabolism of brain areas involved with descending pain modulation, pain perception (i.e., anterior cingulate cortex) and sensory integration (i.e., thalamus). However, based on the fact that only a few studies investigated structural and glucose metabolic changes in chronic SCI subjects with NP, the underlying mechanisms that accompany this condition remains to be further elucidated. Future cross-sectional or longitudinal studies that aim to disentangle NP related to SCI may benefit from stricter constraints in subject cohorts, controlled subgroups, improved pain phenotyping, and implementation of multimodal approaches to discover sensitive biomarkers that profile pain and optimize treatment in SCI subjects with NP.

## Introduction

### Rationale

Spinal cord injury (SCI) has severe consequences for the individual, commonly causing distinct motor and sensory deficits below the level of lesion, attributed to the damage of the corresponding efferent and afferent neural pathways. Disruption of the somatosensory system after SCI can lead to debilitating chronic neuropathic pain (NP) prevalent in 53% of subjects (1) with a third reporting it to be severe (2). NP is defined by the International Association for the Study of Pain (IASP) as “pain arising as a direct consequence of a lesion or disease affecting the (central) somatosensory system” (3, 4). The pathophysiology of NP involves a complex interaction of neuronal changes, inflammation, glial–neuron interactions, supraspinal and spinal sensitization, and alterations in endogenous pain modulation [see reviews: (5–9)]. Consequences of these mechanistic changes are chronic alterations of nociceptive pathways and pain processing regions within the central nervous system, i.e., spinal cord, brainstem, and the brain. Indeed, current neuroimaging studies investigating chronic pain conditions, i.e., fibromyalgia, complex regional pain syndrome, and chronic low back pain have observed changes in brain structure (10, 11), resting-state connectivity (12–14), and metabolic function (15). Furthermore, gray matter volume in chronic low back pain (16) and connectivity changes in fibromyalgia subjects (17), respectively, were shown to be reversed concurrently following pain treatment (16, 17). Together, these studies suggest that changes of structural and functional plasticity within the brain accompany those who experience chronic pain [see reviews: (18, 19)]. However, in cross-sectional studies alone, determining whether the observed brain differences in chronic pain conditions are pre-existing the pain condition, a marker of chronic pain predisposition, or a direct cause of the pain condition itself remains debatable.

Nevertheless, within the last few years, advanced magnetic resonance imaging (MRI) has provided a valuable tool to investigate the structural and/or functional correlates of NP in the brain (5, 18, 20–22). In this context, four MRI methods have been mainly used:

1. Structural imaging including voxel-based morphometry (VBM) and voxel-based cortical thickness (VBCT). VBM is an imaging-based analysis method for quantifying changes in gray and white matter volume (GMV and WMV, respectively) (23). VBCT is an applied surface-based metric to study atrophy (24). Both methods can provide complementary information of alterations in density and thickness of brain areas in diseases (25).
2. Diffusion tensor imaging (DTI) measures the diffusion of water molecules in each image voxel and allows indirect quantification of peripheral and central WM integrity and myelination. In the nervous tissue, physical properties such as neuronal density, axon diameter, fiber bundles, and degree of myelination all affect diffusion metrics (26, 27). Fractional anisotropy (FA) and mean diffusivity (MD) are most commonly used DTI metrics (28–30). FA measures the anisotropy of tissue architecture by the preferential direction of water movement, i.e., water molecules diffuse along the direction of tightly packed axons, whereas MD measures the degree of water diffusion. DTI can also visualize and trace WM tracts indirectly with tractography, which can provide anatomical information on specific fibers of interest (31, 32). Yet, careful interpretation is required as several possible substrates can impact on DTI measures and findings may be heterogenous in different neurological disorders (33).
3. Functional MRI (fMRI) informs about brain function, measured by alterations in the blood oxygen level-dependent signal (34) or by arterial spin labeling (35) that provides a measure of cerebral blood flow. Under task conditions, the hemodynamic response can primarily reflect the local processing of a brain region (36). fMRI can also measure functional connectivity under resting (rsfMRI) or task conditions. This method utilizes the blood oxygen level-dependent signal to define brain areas that have low-frequency synchronous coactivation or resting-state functional connectivity (rsFC).
4. Magnetic resonance spectroscopy (MRS) (37) provides *in vivo* biochemical information including *N*-acetyl aspartate (NAA), myo-inositol (Ins), creatine (Cr), combined measures of glutamate and glutamine (Glx), glutamate (Glu), choline (Cho), and gamma(γ)-aminobutyric acid (GABA). This method allows the determination of metabolite concentrations of certain brain regions and is valuable to investigate underlying biochemical changes that may complement structural and functional observations in clinical populations (38–40).

Next to MRI-based methods, positron emission tomography (PET) can be used to investigate neurochemical changes, i.e., molecular processes, receptor activity, or density alongside blood flow and glucose metabolism in a variety of clinical applications (41–43).

To date, recent reviews and a meta-analysis have summarized the cortical alterations, alongside metabolite and functional changes after SCI [for reviews and meta-analysis, see (44–47)]. In addition, two reviews summarized the impact of deafferentation and chronic pain upon brain reorganization reported with task-based fMRI in animal and human studies (20, 48). However, possibly due to the heterogeneous nature of SCI, i.e., variable injury level and completeness (American Spinal Cord Injury Association Impairment Scale) (49), injury duration, extent of deafferentation (50, 51), and varied clinical characteristics of NP (52), there is limited understanding of the associated changes of brain structure, rsFC, and metabolism in SCI subjects with and without NP.

### Objectives

The overall aim of this systematic review is to determine how current neuroimaging studies have provided understanding of neuroplastic changes that possibly contributes to NP following SCI. A variety of neuroimaging studies with different modalities and parameters have appeared to investigate the changes of brain structure and function that accompany chronic NP following SCI, but a synthesis of the literature is lacking. This review therefore expects to compile and examine the remote brain changes as reported in the current literature in two particular SCI states: (1) SCI without NP and (2) SCI with NP.

### Research Questions

Specific research questions include the following:

1. What are the brain correlates, i.e., structure, functional connectivity, and metabolism that accompany NP after SCI?
2. What have current neuroimaging results revealed regarding the underlying pathophysiological mechanisms of NP following SCI?
3. What do future neuroimaging studies need to consider and account for when investigating NP in SCI subjects?

## Methods

### Study Design and Search Strategy

**Figure 1:**
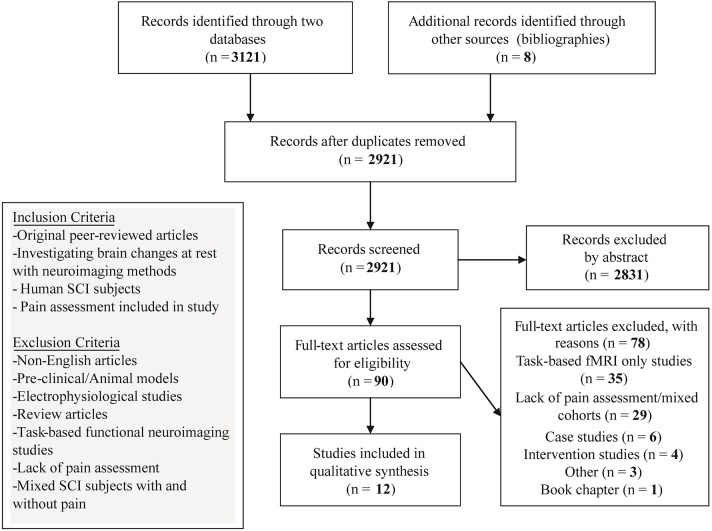
Flow diagrnm of the method followed through the systematic review according to PRISMA standards.

A search of the online databases PubMeD and SCOPUS was conducted on the 1st July 2019 up to 1st August 2019. No publication year criterion was set. The search was performed using a combination of Medical Subject Headings (MeSH) keywords: “Tetraplegia,” “Quadriplegia,” “Paraplegia,” “Spinal Cord Injury,” “Neuropathic Pain,” “Voxel Based Morphometry,” “Voxel Based Cortical Thickness,” “Diffusion Tensor Imaging,” “Diffusion Magnetic Resonance Imaging,” “DTI,” “Resting-state fMRI,” “rs fMRI,” “Resting-State Functional Connectivity,” “Functional Neuroimaging,” “Functional Magnetic Resonance Imaging,” “fMRI,” “Magnetic Resonance Spectroscopy,” and “Positron-Emission Tomography” to identify neuroimaging studies investigating SCI subjects with and without NP. Specifically, MeSH term combinations involved using logic term “OR” between the SCI terms, i.e., Tetraplegia, Quadriplegia etc. while the term “AND” was added to include the neuroimaging method. To optimize the search for identifying articles that investigated the NP condition, an additional “AND” with “Neuropathic Pain” was placed after the neuroimaging method (see Supplementary Table 1). To miss relevant studies, bibliographies of identified studies were also hand searched. One author (VH) initiated the article search utilizing MeSH term combinations and filtered article abstracts for relevancy after removal of duplicates. This yielded a preselection of 90 articles that were further inspected with the inclusion and exclusion criteria for relevancy. Preselected articles (*n* = 90) were additionally checked for inclusion and exclusion criteria by two additional authors (MH and LM) (Figure 1).

### Data Sources, Studies Sections, and Data Extraction

All original studies that investigated supraspinal changes in SCI subgroups with and without NP employing neuroimaging methods were included. Studies that included SCI subjects but lacked pain assessments were excluded. Studies that included pain assessments but did not differentiate SCI subjects into pain subgroups were not included in the qualitative synthesis due to potential confounds in their results. Furthermore, non-English studies, preclinical studies, electrophysiological studies, case studies, intervention studies, review articles, and task-based functional neuroimaging only studies were excluded.

The outcomes extracted and summarized from each study included the following: (1) the modality of the neuroimaging study and subjects' pain assessment implemented in the article; (2) subjects involved, i.e., number, age, chronicity of injury, level of injury, sex, completeness of injury (i.e., Association Impairment Scale A—sensorimotor complete) and presence of NP as assessed by the study's pain characterization (**Table 2**); (3) whether the study implemented a whole brain and/or a region of interest approach for data analysis; and (4) the main findings of brain alterations from comparisons between healthy controls and/or SCI subgroup (**Tables 3**–**5**).

## Results

### Systematic Search Results

Initial search in the two databases and bibliographies yielded 2,921 articles after the removal of duplicate entries. Screening of abstracts derived 90 articles that were manually screened for relevance. Upon further inspection with the inclusion and exclusion criteria, 35 were excluded due to being task-based only fMRI studies; 29 articles included SCI subjects but were excluded due to lacking pain characterization or including mixed cohorts (SCI with and without NP) for the analysis (see Supplementary Table 2); 4 were intervention studies and 6 single case studies were excluded; 1 was excluded due to being a book chapter; and 3 were excluded due to other reasons (Figure 1, Supplementary Table 2).

As a result, 12 articles were included in the synthesis (Table 1). Four articles included sMRI (one study also included task-based fMRI results that were omitted from the qualitative synthesis); one study also included rsfMRI. One article included sMRI, DTI, and PET. One study included DTI only. One study included rsfMRI only. Five articles included MRS (one study included task-based fMRI results that were omitted from the qualitative synthesis). Article findings are summarized and presented based on the comparison between SCI subgroups to healthy controls (**Tables 4**, **5**) and between the two SCI subgroups (with/without NP) (**Table 3**). Demographics of SCI subgroups are summarized in Table 2.

**Table 1:** Overview of articles investigating brain alterations in SCI subjects without or with NP included in qualitative synthesis.

**References**	**Modality**	**HC** ** (*n*)**	**SCI** ** (*n*)**	**Pain characterization**
Chen et al. (53)	sMRI^*^	13	13	VAS at MRI acquisition
Chen et al. (54)	sMRI, rsfMRI	11	11	VAS at MRI acquisition
Jutzeler et al. (55)	sMRI	31	28	EMSCI pain questionnaire, NRS (0–10)
Mole et al. (56)	sMRI	18	30	Below-level NP >1 year, NRS (4–10)
Yoon et al. (57)	sMRI, DTI, PET	10	10	ISCI basic pain dataset, NRS (0–10)
Gustin et al. (58)	DTI	45	23	IASP assessment, NRS (0–10)
Min et al. (59)^§^	rsfMRI	18	18	VAS (0–100) at MRI acquisition
Widerström-Noga et al. (60)	MRS	24	54	MPI-SCI, NRS (0–10), pain diary
Gustin et al. (61)	MRS^*^	21	22	IASP SCI pain taxonomy, VAS (0–10)
Widerström-Noga et al. (62)	MRS	24	68	MPI-SCI, NRS (4–10)
Stanwell et al. (63)	MRS	10	10	Pain interview and assessment
Pattany et al. (64)	MRS	10	16	Pain interview, drawings, NRS (0–10)

*Summary of articles included in qualitative synthesis. Details include neuroimaging modality, number of SCI subjects with or without NP, number of healthy controls, and NP characterization used to differentiate or characterize SCI subjects. Ranges from 0 to 10 or 0 to 100 are commonly used to depict “no pain” to “worst pain imaginable” for NRS and VAS*.

*DTI, diffusion tensor imaging; EMSCI, European Multicenter Study about Spinal Cord Injury; HC, healthy controls; IASP, International Association for the Study of Pain; ISCI, International Spinal Cord Injury; ISCIP, International Spinal Cord Injury Pain Classification; MPI, multidimensional pain inventory; MRI, magnetic resonance imaging; MRS, magnetic resonance spectroscopy; NP, neuropathic pain; NRS, numerical rating scale; PET, positron emission tomography; rsfMRI, resting-state functional MRI; SCI, spinal cord injury; sMRI, structural MRI; VAS, Visual Analog Scale*.

* *Study also included task-based fMRI results omitted in qualitative synthesis*.

§ *Study did not identify whether pain was nociceptive or neuropathic*.

**Table 2:** Demographics of SCI subgroups included in the study.

**References**	**SCI** ** subjects**	***n***	**Age** ** (years)**	**Sex** ** (m/f)**	**LOI**	**AIS** ** (A–E)**	**TSI** ** (d, wks, mths, yrs)**	**Averaged** ** pain intensity**
Chen et al. (53)	SCI-NP	0	–	–	–	–	–	–
	SCI-no NP	13	51.3 ± 6.4	10/3	C3–C8	2B, 2C, 9D	11.7 ± 15.3 d	0
Chen et al. (54)	SCI-NP	0	–	–	–	–	–	–
	SCI-no NP	11	50 ± 4.9	9/2	C3–C8	1B, 2C, 8D	10.0 ± 7.9 d	0
Jutzeler et al. (55)	SCI-NP	13	46.9 ± 11.4	11/2	C2–L3	5A, 2B, 6D	12.8 + 8.4 yrs	4.0 ± 2.1
	SCI-no NP	15	45.9 ± 12.8	15/0	C4–T12	6A, 3B, 2C, 4D	12.2 ± 7.7 yrs	0
Mole et al. (56)	SCI-NP	18	51.3 ± 7.9	n/a	C5–T5	n/a	11.1 ± 8.5 yrs	6.0 ± 1.6
	SCI-no NP	12	54.3 + 16.9	n/a	C5-T5	n/a ^†^A-D	17.7 ± 11.4 yrs	0
Yoon et al. (57)	SCI-NP	10	39.8 ± 6.1	7/3	C4–T11	7A, 3B	18.4 ± 6.1 mths	7.6 ± 0.5
	SCI-no NP	0	–	–	–	–	–	–
Gustin et al. (58)	SCI-NP	12	48.0 ± 4.0^*^	n/a	C8–T10	12A	16.0 ± 5 yrs^*^	4.3 ± 0.4
	SCI-no NP	11	38.0 ± 3.0^*^	n/a ^†^19/4	T3–T10	11A	13 ± 2 yrs^*^	0
Min et al. (59)^§^	SCI-NP^§^	18	57.7 ± 11.9	12/6	C2–C7	5C, 13D	49.8 ± 33.7 wks	46.6 ± 23.3
	SCI-no NP	0	–	–	–	–	–	–
Widerström-Noga et al. (60)	SCI-NP							
	SCI-HNP	19	43.0 ± 12.5	16/3	n/a	6A, 13B–D^**^	12.0 ± 9.7 yrs	6.4 ± 1.6
	SCI-LNP	35	35.7 ± 12.4	28/7	n/a	24A, 11B–D^**^	13.1 ± 9.7 yrs	1.6 ± 1.5
	SCI-no NP	0	–	–	–	–	–	–
Gustin et al. (61)	SCI-NP	12	57.0 ± 4.0^*^	8/4	T3–T12	12A	n/a	3.6 ± 0.8
	SCI-no NP	10	50.0 ± 4.0^*^	8/2	T3–T10	10A	n/a	0
Widerström-Noga et al. (62)	SCI-NP							
	SCI-HNP	19	40.4 ± 11.8	14/5	C–S5^**^	9A, 10B–D^**^	12.0 ± 9.9 yrs	~4.1 ± 0.8
	SCI-LNP	31	37.5 ± 13.4	26/5	C–S5^**^	17A, 14B–D^**^	10.6 ± 9.1 yrs	~3.1 ± 1.0
	SCI-no NP	18	36.8 ± 11.0	14/4	C-S5^**^	14A, 3B–D^**^^,^ 1 n/a	16.2 ± 9.5 yrs	–
Stanwell et al. (63)	SCI-NP	5	n/a	n/a	n/a	5A	63.6 ± 49.6 mths	n/a
	SCI-no NP	5	n/a ^†^36.4 ± 10.4	n/a	n/a ^†^T	5A	58.2 ± 59.7 mths	n/a
Pattany et al. (64)	SCI-NP	7	46.2 ± 16.2	7/0	C8–L3	n/a	7.6 ± 6.3 yrs	5 <^**^
	SCI-no NP	9	34.8 ± 10.0	9/0	C4–L3	n/a	11.3 ± 9.6 yrs	0

*Summary of demographics for SCI subgroups as described in each study. Study order is based on neuroimaging modality as reported in Table 1, starting with structural, DTI, rsfMRI, then MRS. Averaged pain intensity: ranges from 0 to 10 or 0 to 100 are commonly used to depict “no pain” to “worst pain imaginable” on a numerical rating scale and visual analog scale, respectively*.

*AIS, ASIA Impairment Scale (A, sensorimotor complete; B, motor complete, sensory incomplete; C and D, sensory motor incomplete; E, normal); C, cervical level; d, days; f, female; L, lumbar level; LOI, level of injury; m, male; mths, months; n/a, not available; NP, neuropathic pain; S, sacral level; SCI, spinal cord injury; SCI-HNP, SCI subjects with high NP; SCI-LNP, SCI subjects with low NP; SCI-NP, SCI subjects with NP; SCI-no NP, SCI subjects without NP; T, thoracic level; TSI, time since injury; wks, weeks; yrs, years*.

*Traumatic SCI*.

* *Standard error of mean*.

** *Unspecified information*.

*~Multidimensional pain inventory—SCI version subscale: pain severity*.

§ *Study did not identify whether pain was nociceptive or neuropathic*.

† *Mean and standard deviation of whole SCI cohort*.

### Pain Assessments Implemented in Included Articles

Within these studies, a range of pain assessments were used to characterize the presence of pain in SCI subjects (Table 1). The presence of NP in SCI subjects were characterized in nine studies with interviews, pain drawings, and questionnaires (55–58, 60–64) (Table 1). Three studies utilized a visual analog scale (VAS) on the day of MRI acquisition to assess pain; NP assessment was not mentioned (53, 54, 59). Seven studies characterized pain intensity with a numerical rating scale (NRS) (55–58, 60–64); two of these studies used NRS on the day of MRS acquisition (60, 64). One study assessed pain intensity but did not report the scale of which they used (63). Three studies characterized pain intensity using a pain diary for a week (with VAS or NRS) before the day of MRI/MRS acquisition (56, 58, 61). Two studies included SCI subjects with NP who had a minimum pain rating of 4 (out of 10) (56, 62).

### Neuroimaging Studies Investigating NP Within SCI

Based on the eligible articles, eight studies investigated the impact of NP within SCI subjects (55, 56, 58, 60–64) (Table 2). Two studies did not differentiate their SCI subjects as their whole cohort had some degree of NP (57, 59) (Tables 1, 2).

Volumetric differences between chronic SCI subjects with and without NP were reported in two studies (55, 56). In SCI subjects with NP, one study observed increased GMV of the left anterior cingulate cortex (ACC) and right M1 with decreased GMV of the right S1 and bilateral thalamus (55). This study also showed a positive correlation with pain intensity and GMV of M1 (55). Another study showed decreased GMV of S1 and WMV of S2 in SCI subjects with NP (56) alongside a negative correlation with pain intensity and GMV of S1 (56).

Only one DTI study investigated microstructural differences between chronic SCI subjects with and without NP (58). This study observed decreased MD of the ventral pons to midbrain region, alongside increased MD of prefrontal, premotor and parietal cortices, anterior insula, thalamus, and amygdala in SCI subjects with NP (58). In addition, MD values of the anterior insular, prefrontal, premotor, and parietal cortices were positively correlated with pain intensity, whereas MD values of the amygdala and thalamus were negatively correlated with pain intensity (58).

Four studies investigated metabolite concentration differences between SCI subjects with and without NP (60–64). Two studies observed decreased NAA/Cr and GABA/Cr ratios (61) and decreased absolute Glx and Glx/Ins ratios (62) in the thalamus of SCI subjects with NP. One study reported decreased Glx and Glx/Ins ratios in the ACC of SCI subjects with NP compared to those without NP (62). This study also investigated differences between SCI subjects with differing amounts of NP intensity, observing increased Ins, Cr, and Cho with decreased NAA/Ins and Glx/Ins ratios in the ACC of SCI subjects with greater NP intensity compared to those with a lower NP intensity (62). Another study observed decreased NAA/Ins and Glx/Ins within the thalamus of SCI subjects with greater NP intensity compared to those with a lower NP intensity (60). One study using wavelet-based statistics of MRS data identified mean spectral differences within the prefrontal cortex (PFC) and ACC (but not the thalamus) between SCI subjects with and without NP (63). These differences may be related to a variety of metabolite concentrations, i.e., NAA, GABA, Glu, Ins, and Asp (63) (Table 3).

**Table 3:** Neuroimaging studies investigating NP within the SCI cohort.

**References**	**Modality**	**Region of interest(s)**	**Statistical correction**	**Main findings**
Jutzeler et al. (55)	sMRI	Whole brain; M1, S1, S2, PMC, insula, thalamus, ACC as regions of interests	*p* < 0.05 FWE correction	SCI-NP vs. SCI-no NP: *(Whole brain):*↔ GMV *(Regions of interest):* ↑ GMV in L ACC and R M1 Pain intensity correlated positively with GMV in R M1 ↓ GMV in R S1 and bilateral thalamus.
Mole et al. (56)	sMRI	Whole brain: M1, S1, thalamus, L posterior cingulate, R insula as regions of interests	*p* < 0.001 uncorrected; *p* < 0.05 FWE correction	SCI-NP vs. SCI-no NP: ↓ GMV in bilateral S1. Pain intensity correlated negatively with GMV in S1 ↓ WMV deep to S2
Min et al. (59)^§^	rsfMRI	Bilateral M1, SMA, S1, S2, BG, dlPM, vlPM	*p* < 0.05, *k* = 64 FDR correction	SCI-pain vs. SCI-no pain: n/a
Yoon et al. (57)	sMRI DTI PET	Whole brain	sMRI and PET: *p* < 0.001 uncorrected, *p* < 0.05 SVC with 10 mm spheres; DTI: *p* < 0.05 TFCE	SCI-NP vs. SCI-no NP: n/a
Gustin et al. (58)	DTI	Whole brain	*p* < 0.005 uncorrected, *k =* 20	SCI-NP vs. SCI-no NP: ↓ MD of ventral pons to midbrain. ↑ MD of R PPC, R dorsolateral PFC, L anterior insula, mOFC, PMC, L amygdala, and R ventroposterior thalamus. MD values of dorsolateral PFC, PPC, anterior insula and PMC were positively correlated with pain intensity. MD values of amygdala and ventroposterior thalamus were negatively correlated with pain intensity. ↔ FA.
Widerström- Noga et al. (60)	MRS	Thalamus	Independent *t* tests *p* < 0.05	SCI-NP high pain vs. low pain:↓ NAA/Ins and Glx/Ins
Gustin et al. (61)	MRS	Thalamus	Independent *t* tests *p* < 0.05	SCI NP vs. SCI no NP:NAA/Cr and GABA/Cr
Widerström-Noga et al. (62)	MRS	ACC	Independent *t* tests *p* < 0.05	SCI-NP high pain vs. low pain:Ins, Cr and Cho ↓ NAA/Ins and Glx/Ins SCI-NP high pain vs. SCI no NP: ↓ Glx ↓ Glx/Ins
Stanwell et al. (63)	MRS	Thalamus, PFC, ACC	Wavelet-based significant testing *p* < 0.05	SCI-NP vs. SCI-no NP: *PFC:* Mean spectral differences, possible contributions: NAA, Glu, Glx, Cho, taurine and GABA. *ACC:* Mean spectral differences, possible contributions: Ins and Asp. *Thalamus:* not significant
Pattany et al. (64)	MRS	Thalamus	Post hoc *t*-tests *p* < 0.05	SCI-NP vs. SCI-no NP: ↓ NAA and NAA/Ins. Pain intensity correlated negatively with NAA levels. Pain intensity correlated positively with Ins levels

*Results of studies investigating differences between SCI-NP and SCI-no NP subjects unless stated otherwise. Study order is based on neuroimaging modality as reported in Table 1, starting with structural, DTI, rsfMRI, then MRS. ACC, anterior cingulate cortex; Asp, aspartate; BG, basal ganglia; CC, corpus callosum; Cho, choline; Cr, creatine; CST, corticospinal tract; DTI, diffusion tensor imaging; dlPM, dorsolateral premotor cortex; FA, fractional anisotropy; FC, functional connectivity; FDR, false-discovery rate; FWE, family-wise error; GABA, gamma (γ)-aminobutyric acid; Glu, glutamate; Glx, glutamate and glutamine; GMV, gray matter volume; Ins, Myo-inositol; k, minimum cluster size; L, left; M1, motor cortex; MD, mean diffusivity; mFG, medial frontal gyrus; mOFC, medial orbital frontal cortex; MRS, magnetic resonance spectroscopy; NAA, N-acetyl aspartate; PET, positron emission tomography; PFC, prefrontal cortex; PMC, premotor cortex; PPC, posterior parietal cortex; R, right; rsfMRI, resting-state fMRI; S1, somatosensory cortex 1; S2, somatosensory cortex 2; SCI, spinal cord injury; SLF, superior longitudinal fasciculus; SMA, supplementary motor area; sMRI, structural MRI; SCI NP, SCI subjects with NP; SCI no NP, SCI subjects without NP; SVC, small volume correction; TFCE, threshold-free cluster enhancement; vlPM, ventrolateral premotor cortex; WMV, white matter volume*.

*↔ No significant changes; ↑ significant increase; ↓ significant decrease*.

### Neuroimaging Studies Investigating SCI Subjects With NP Compared to Healthy Controls

Eight articles compared SCI subjects with NP to healthy controls (Table 4), showing a variable impact of SCI accompanied by NP upon brain structure, bidirectional microstructural, and connectivity changes and alterations in metabolite concentrations compared to healthy cohorts.

**Table 4:** Neuroimaging studies investigating SCI subjects with NP compared to healthy controls.

**References**	**Modality**	**Region of interest(s)**	**Statistical correction**	**Main findings**	
Jutzeler et al. (55)	sMRI	Whole brain; M1, S1, S2, PMC, insula, thalamus and ACC as regions of interests	*p* < 0.05 FWE correction	SCI-NP vs. controls: ↑ GMV in ACC. ↓ GMV in Thalamus	
Mole et al. (56)	sMRI	Whole brain: M1, S1, thalamus, L posterior cingulate, R insula as regions of interests	*p* < 0.001 uncorrected; *p* < 0.05 FWE correction	SCI-NP vs. controls: ↔GMV. ↓ WMV of bilateral pyramids, L medial cuneus, deep to L S2 and R posterior corona radiata	
Min et al. (59)^§^	rsfMRI	M1, SMA, S1, S2, BG, dlPM, vlPM	*p* <0.05, *k* = 64 FDR correction	SCI-pain vs. controls: ↓ FC: •R M1—R S1, R S2 •R S1—L S1, R S2, L S2 •L S1—R S2 •R S2—R dlPM	↑ FC: •R M1—R SMA, L SMA •L BG—L S2
Yoon et al. (57)	sMRI DTI PET	Whole brain	sMRI and PET: *p* < 0.001 uncorrected, *p* < 0.05 SVC with 10 mm spheres; DTI: *p* < 0.05 TFCE	SCI-NP vs. controls: ↓ GMV in L mFG, R ACC and bilateral anterior insula ↓ MD: splenium and body of CC, R SLF, CST regions, thalamocortical tract, superior parietal white matter, R pre and post central, bilateral superior frontal and middle frontal area, cerebral peduncle, anterior corona radiata and internal capsule. ↔ FA ↓ metabolism in L middle frontal gyrus and R mFG	
Gustin et al. (58)	DTI	Whole brain	*p* < 0.005 uncorrected, *k =* 20	SCI-NP vs. controls: ↑ MD of dorsolateral PFC, PPC and PMC ↓ MD in ventroposterior thalamus, amygdala and ventral pons ↔ FA	
Widerström- Noga et al. (60)	MRS	Thalamus	Independent *t* tests *p* < 0.05	SCI-NP high pain vs. controls: ↓ NAA/Ins and Glx/Ins SCI-NP low pain vs. controls: ↔ metabolite concentrations	
Gustin et al. (61)	MRS	Thalamus	Independent *t* tests *p* < 0.05	SCI-NP vs. controls: ↓ NAA/Cr and GABA/Cr	
Widerström-Noga et al. (62)	MRS	ACC	Independent *t* tests *p* < 0.05	SCI -NP high pain vs. controls: ↑ Glx/Ins SCI-NP low pain vs. controls: n/a	
Stanwell et al. (63)	MRS	Thalamus, PFC, and ACC	Wavelet-based significant testing *p* < 0.05	SCI-NP vs. controls: n/a	
Pattany et al. (64)	MRS	Thalamus	Post hoc *t* tests *p* < 0.05	SCI-NP vs. controls: ↔ NAA and NAA/Ins	

*Results of studies investigating differences between SCI-NP subjects and healthy controls unless stated otherwise. Study order is based on neuroimaging modality as reported in Table 1, starting with structural, DTI, rsfMRI, then MRS*.

*ACC, anterior cingulate cortex; BG, basal ganglia; CC, corpus callosum; Cr, creatine; CST, corticospinal tract; DTI, diffusion tensor imaging; dlPM, dorsolateral premotor cortex; FA, fractional anisotropy; FC, functional connectivity; FDR, false-discovery rate; FWE, family-wise error; GABA, gamma (γ)-aminobutyric acid; Glx, glutamate and glutamine; GMV, gray matter volume; Ins, myo-inositol; k, minimum cluster size; L, Left; M1, motor cortex; MD, mean diffusivity; mFG, medial frontal gyrus; MRS, magnetic resonance spectroscopy; NAA, N-acetyl aspartate; PET, positron emission tomography; PFC, prefrontal cortex; PMC, premotor cortex; PPC, posterior parietal cortex; R, right; rsfMRI, resting-state fMRI; S1, somatosensory cortex 1; S2, somatosensory cortex 2; SCI, spinal cord injury; SLF, superior longitudinal fasciculus; SMA, supplementary motor area; sMRI, structural MRI; SCI NP, SCI subjects with NP; SCI no NP, SCI subjects without NP; SVC, small volume correction; TFCE, threshold-free cluster enhancement; vlPM, ventrolateral premotor cortex; WMV, white matter volume*.

*↔ No significant changes; ↑ significant increase; ↓ significant decrease*.

Compared to healthy controls, volumetric changes in SCI subjects with NP were reported in two studies (56, 57); one study did not report this comparison (55). One study with 18 chronic SCI subjects with NP observed no GMV changes but decreased WMV in the bilateral pyramids, medial cuneus, secondary somatosensory cortex (S2), and posterior corona radiata (56).

Another study using multimodal imaging in 10 SCI subjects with NP observed decreased GMV in the medial frontal gyrus, ACC, and anterior insula (57). This study also observed decreased glucose metabolism in the frontal areas (57) and widespread decreases in MD in white matter fibers (i.e., corticospinal tract, thalamocortical tract, and corpus callosum) as well as in frontal regions and sensorimotor areas (57). No changes in FA were observed (57) (Table 4).

Microstructural changes were reported in another study with 12 chronic SCI subjects with NP compared to healthy controls (58). This study observed increased MD in the prefrontal, parietal, and premotor cortices; decreased MD in the thalamus, amygdala, and ventral pons; and no changes in FA (58) (Table 4).

Bidirectional connectivity changes were reported in 18 SCI subjects with a varying amount of pain as identified on the VAS (59). Decreases in rsFC between interhemispheric primary motor cortex (M1), premotor cortex, S1, and S2 were observed alongside increased rsFC of M1 to supplementary motor area and the basal ganglia and S2 (Table 4) (59). Although unclear whether the pain was neuropathic or nociceptive in this study, no correlations between rsFC changes and pain severity were reported (59).

Metabolite changes in the thalamus and ACC between chronic SCI subjects with NP and healthy controls were investigated in four studies (58, 60, 62, 64) (Table 4). Two studies that investigated the thalamus observed decreases in NAA/Ins and NAA/Cr ratios (60, 61), decreased Glx/Ins ratio (60), and GABA/Cr ratio (61). One study investigating SCI subjects with a mild NP showed no significant metabolite alterations in the thalamus compared to healthy controls (60); another study also found no changes in metabolite concentrations in the thalamus (64). One study reports decreased Glx/Ins ratios within the ACC of SCI subjects with more severe NP compared to healthy controls (62).

### Neuroimaging Studies Investigating SCI Subjects Without NP Compared to Healthy Controls

Based on the eligible articles, SCI subjects without NP show variable alterations in brain structure and decreased connectivity of frontal and visual areas and metabolite concentrations in the thalamus compared to the healthy condition (53–56, 58, 61, 62, 64) (Table 5).

**Table 5:** Neuroimaging studies investigating SCI subjects without NP compared to healthy controls.

**References**	**Modality**	**Region of interest(s)**	**Statistical correction**	**Main findings**
Chen et al. (53)	sMRI	Whole brain; S1, M1 and thalamus as region of interests	*p* < 0.05 cluster-level corrected	SCI-no pain vs. controls: ↓ GMV in L superior parietal lobule ↓ WMV in R temporal lobe, R occipital lobe and R calcarine gyrus
Chen et al. (54)	sMRI rsfMRI	Whole brain	sMRI: *p* < 0.0001 uncorrected, *p* < 0.05 cluster-level FWE correction; rsfMRI: *p* < 0.05 *k* = 30 (Monte Carlo simulation)	SCI-no pain vs. controls:↓ GMV in L hippocampus, L parahippocampal gyrus, R superior and middle frontal gyrus↔ WMV↓ ALFF in L OFC↓ intranetwork FC in L middle occipital gyrus
Jutzeler et al. (55)	sMRI	Whole brain; M1, S1, S2, PMC, insula, thalamus, and ACC as regions of interests	*p* < 0.05 FWE	SCI-no NP vs. controls:↓ GMV in ACC and M1
Mole et al. (56)	sMRI	Whole brain:M1, S1, thalamus, L posterior cingulate, R insula as regions of interests	*p* < 0.001 uncorrected; *p* < 0.05 FWE correction	SCI-no NP vs. controls: ↑ GMV in bilateral S1 and L lateral cuneus ↓ WMV of bilateral pyramids and posterior corona radiata
Gustin et al. (58)	DTI	Whole brain	*p* < 0.005 uncorrected, *k =* 20	SCI-no NP vs. controls: ↓ FA of PPC-to-midbrain fibers ↓ MD of anterior insula, PPC and dorsolateral PFC, nucleus accumbens, ventroposterior thalamus and PMC
Gustin et al. (61)	MRS	Thalamus	Independent *t* tests *p* < 0.05	SCI-no NP vs. controls: No significance
Widerström-Noga et al. (62)	MRS	ACC	Independent *t* tests *p* < 0.05	SCI-no NP vs. controls: No significance
Stanwell et al. (63)	MRS	Thalamus, PFC, and ACC	Wavelet-based significant testing *p* < 0.05	SCI-no NP vs. controls: n/a
Pattany et al. (64)	MRS	Thalamus	Post hoc *t* tests *p* < 0.05	SCI-no NP vs. controls:↔ NAA↑ NAA/Ins↓ Ins

*Results of studies investigating differences between SCI-no NP subjects and healthy controls unless stated otherwise. Study order is based on neuroimaging modality as reported in Table 1, starting with structural, DTI, rsfMRI, then MRS*.

*ACC, anterior cingulate cortex; ALFF, amplitude of low-frequency fluctuations; DTI, diffusion tensor imaging; FA, fractional anisotropy; FWE, family-wise error; GMV, gray matter volume; Ins, myo-inositol; k, minimum cluster size; L, left; M1, motor cortex; MD, mean diffusivity; mFG, medial frontal gyrus; MRS, magnetic resonance spectroscopy; NAA, N-acetyl aspartate; PFC, prefrontal cortex; PMC, premotor cortex; PPC, posterior parietal cortex; R, right; rsfMRI, resting-state fMRI; S1, somatosensory cortex 1; S2, somatosensory cortex 2; SCI, spinal cord injury; sMRI, structural MRI; NP, neuropathic pain; SCI NP, SCI subjects with NP; SCI no NP, SCI subjects without NP; WMV, white matter volume*.

*↔ No significant changes; ↑ significant increase; ↓ significant decrease*.

Three studies reported a range of volumetric changes (54–56). Decreased GMV were observed in the left superior parietal lobule (53), left hippocampus, and superior and middle frontal gyrus (54). Increased GMV of bilateral primary somatosensory cortex (S1), left cuneus (56), and ACC (55) were also observed in SCI subjects without NP compared to healthy controls. Decreased WMV were observed in the right temporal and occipital areas (53) alongside the pyramidal region and posterior corona radiata (56) in SCI subjects without NP. One study did not observe any WMV differences (54).

Microstructural brain changes in SCI subjects without NP was reported in one study (58). Eleven chronic SCI subjects without NP showed decreased FA of fibers connecting the parietal cortex to the midbrain and decreased MD of the premotor and PFC, parietal region, anterior insula, nucleus accumbens, and ventroposterior thalamus (58).

Connectivity changes in acute SCI subjects without pain were reported in one study (54). This study observed decreased amplitude of low-frequency fluctuations in the left orbitofrontal cortex, which correlated negatively with subjects' motor scores alongside decreased intranetwork rsFC of the left middle occipital gyrus in the medial visual network (54) (Table 5).

Metabolite concentrations of SCI subjects without NP were reported in three studies (61, 62, 64). Two studies observed no differences in the ACC or thalamus (61, 62) in SCI subjects without NP compared to controls. In contrast, one study reported increased NAA/Ins ratio and decreased Ins in the thalamus of nine chronic paraplegic SCI subjects without NP compared to controls (64) (Table 5).

## Discussion

The primary purpose of this review was to examine the results of current studies that investigated the correlates of chronic NP in brain structure, function, and metabolite changes following SCI. With the adoption of strict exclusion and inclusion criteria in this review (Figure 1, Table 1), current neuroimaging studies report a range of changes in SCI subjects with NP dependent on the comparison to particular cohorts, i.e., to healthy controls or SCI subjects without NP (Tables 3, 4). Some studies report changes in metabolite concentrations within sensory and pain-related regions of SCI subjects with NP compared to those without (Table 3). In addition, a couple of studies report bidirectional volumetric changes, and there remains little evidence of microstructural changes in SCI subjects with NP (Table 3). Moreover, in comparison to healthy controls, there remains limited evidence of volumetric, microstructural, and connectivity changes but a range of alterations in metabolite concentrations in SCI subjects with NP (Table 4). Furthermore, the few studies included here report bidirectional volumetric changes in SCI subjects without NP, and there is limited evidence of microstructure and metabolite alterations in these subjects (Table 4).

### NP Following SCI Is Associated With Changes in Brain Metabolites and Structure

**Figure 2:**
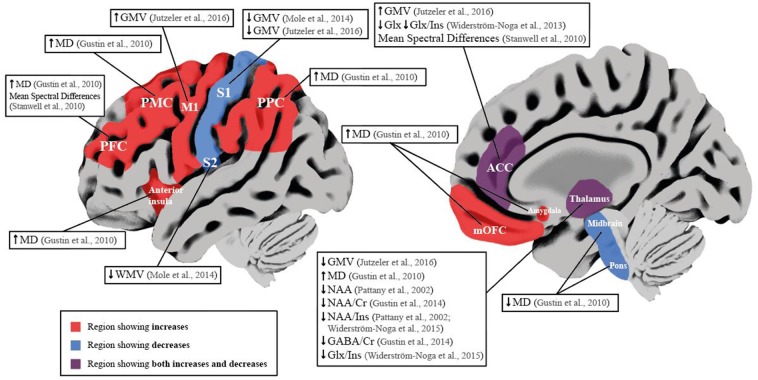
Schematic overview of neuroimaging studies reporting brain differences in SCI subjects with NP compared to SCI subjects without NP. Red brain areas indicate region reported to be affected in articles included in the qualitative synthesis. Labeled boxes describe the type of alteration reported in the brain region. ACC, Anterior Cingulate Cortex; Cr, Creatine; GABA, y-aminobutyric acid; Glx, Glutamate and Glutamine; GMV, Gray Matter Volume; Ins, Myo-inositol; Ml, Prinmy Motor Cortex; MD, Mean Diffusivity; mOFC, Medial Orbitofrontal Cortex; NAA, N-acetyl aspattate; PFC, Prefrontal Cortex; PMC, Premotor Cortex; PPC, Posterior Parietal Cortex; Sl, Primary Somatosensory Cortex; S2, Secondary Somatosensory Cortex; WMV, White Matter Volume. ↓ Significant decrease; ↑ Significant increase.

Current studies that investigated the influence of NP within SCI show primarily metabolite concentration changes in sensory-pain-related regions (i.e., thalamus and ACC) and bidirectional changes in brain volume and microstructure (Table 3, Figure 2).

Within the thalamus of SCI subjects with NP, decreases in NAA ratios to Ins or Cr could be indicative of a loss of neuronal density and dysfunction of inhibitory neurons (61, 64). As decreases in NAA/Cr ratio were positively correlated with the reduction in GABA/Cr ratios, this supports a disruption of normal inhibitory function in SCI subjects with NP compared to those without (61). In addition, Pattany et al., showed negative and positive correlations of NAA and Ins with greater pain intensity, respectively, suggesting neuronal and glial changes and its involvement with pain intensity (64). Moreover, Widerström-Noga et al., reported a decrease in NAA/Ins ratio and Glx/Ins ratio in SCI subjects with intense NP compared to those with mild NP, suggesting a contribution of lower glutamatergic metabolism, increased glial proliferation, or hypertrophy in the thalamus of SCI subjects with more intense NP (60). Similar decreases in NAA/Ins ratio were reported within the ACC in SCI subjects with intense NP compared to those with mild NP (62). This possibly indicates a combination of neuronal dysfunction and gliosis, as absolute concentrations of NAA were not significantly different in SCI subjects with intense NP compared to controls or SCI subjects without NP (62) (Table 3). Furthermore, compared to SCI subjects without NP, SCI subjects with intense NP showed decreased Glx and Glx/Ins ratio, suggesting reduced synaptic activity which may explain the greater amount of emotional distress in this cohort (62), as the ACC is involved in the affective component of pain and descending pain modulation (5, 65–67). To support this view, an earlier study also concluded that mean spectral differences of MRS data within the ACC and PFC delineated SCI subjects with and without NP (63). Structural changes in SCI subjects with NP may be bidirectional, i.e., decreases in GMV in S1 and increases in GMV in M1 both correlate positively with higher pain intensity (55, 56) (Table 3). Decreases in GMV of pain-related regions are often attributed to maladaptive structural plasticity (68–70), whereas increases in GMV reflect preserved structure (also known as “persistent representation”), which correlates to pain intensity (71). In chronic pain conditions, decreases in GMV in sensory-pain-related areas can stem from a variety of factors, i.e., nociceptive input, neurodegeneration, and vascular changes (68, 72, 73). Increases in GMV could be attributed to dendritic branching, neurogenesis, and axonal sprouting (74), and indeed, in subjects with SCI, it has been shown that reorganization of S1 may be attributed to lateral dendritic growth (75). Such bidirectional changes have been previously shown in functional studies in SCI subjects with NP, either reporting increased cortical reorganization (“maladaptive cortical plasticity”) or no change in cortical reorganization (“persistent representation”) correlating positively with higher pain intensity (20, 76). These findings are also reported in functional studies of deafferentated subjects with phantom limb pain (71, 77), and a computational study suggests that these two models are driven by the same underlying mechanism, i.e., abnormal increase in spontaneous activity of deafferented nociceptive pathways (18, 78). It is therefore possible that similar processes occur in SCI subjects with NP reflected in the opposing volumetric changes. One study observed bidirectional microstructural changes in areas involved with spatial cognition, sensory, motor, and affective-motivational functions of SCI subjects with NP (58) (Table 3). Increases in MD were positively correlated with pain intensity in the prefrontal cortex and anterior insula (58), indicating microstructural alterations in regions involved with the cognitive and emotional aspects of pain (58). Decreased MD was positively correlated with pain intensity in the thalamus and amygdala (58), again indicating microstructural changes can be related to pain phenotype. Overall, this may suggest microglial changes that mediate the inflammatory process in SCI subjects with NP as activated microglia are higher in numbers with different phenotypes (larger cell bodies and thicker and shorter processes) (79). It is recognized that activated microglia may contribute to the chronification of NP through hyperexcitability of the sensory neuroaxis and disruption of GABAergic transmission after SCI (80–82).

### Brain Changes Accompanying Both SCI and NP

Although the effects of SCI-related NP remain difficult to disentangle without subgroups of SCI subjects without NP, some studies report the associated brain changes in SCI and NP by comparing these subjects directly to healthy controls (Table 4).

A couple of studies investigated volumetric changes between SCI subjects with NP to controls. Mole et al., observed only WMV decreases in regions of projection pathways with no changes in GMV (56), whereas Yoon et al., reported GMV decreases within the medial frontal gyrus, ACC, and anterior insula (57). These limited results may suggest an impact of SCI and NP upon affective-motivational, pain modulatory, and cognitive function, as Yoon et al., also reported decreased glucose metabolism of the frontal areas within the same cohort (57). Furthermore, microstructural alterations were reported in a couple of studies and may indicate bidirectional changes in water diffusion that accompany SCI and NP (57, 58). Decreased MD was observed in multiple brain regions including the thalamus, amygdala, sensorimotor cortices, and brainstem regions of SCI subjects with NP (57, 58) (Table 4). These decreases could be due to changes in underlying cell properties (i.e., shape and density) and increased extracellular matrix (83). In contrast, both increased MD (58) and decreased MD (57) was observed in the frontal and parietal cortices (Table 4), which suggests glial/neuronal alterations or inflammation (i.e., edema) (30, 83, 84) that accompanies regions involved with emotional or spatial cognition following SCI and NP. Both studies did not report any changes in FA, suggesting that axonal integrity may not be compromised in SCI and NP at the supraspinal level (57, 58). Connectivity changes within the sensory and motor cortices were observed in one study of 18 incomplete SCI subjects with a varying degree of pain (59). In particular, rsFCs between motor regions were significantly increased while rsFC decreases were seen between sensory regions (59) (Table 4). These results suggest that the impact of incomplete SCI may result in cortical rsFC changes due to damage to primary afferents, loss of sensory feedback, and recruitment of motor regions to compensate for the motor deficit (59). Although the authors suggested that pain was not a contributing factor to rsFC changes as no correlations were observed with pain intensity (59), the presence of pain itself cannot be fully overlooked. A few studies observed changes in metabolite concentrations in sensory-pain-related regions of chronic SCI subjects with NP, i.e., ACC and thalamus (60–62). Compared to controls, SCI subjects with NP showed decreased NAA, Glx, and GABA ratios within the thalamus (60, 61) (Table 4), suggesting lower glutamatergic metabolism, loss of neuronal density, and dysfunction of inhibitory neurons that accompanies SCI and NP (38, 60, 61). Although one study did not observe changes in thalamus metabolite concentrations (64), this may be due to the small number of SCI subjects with NP (*n* = 7) (Tables 3, 4). Changes in metabolite concentrations within the ACC were observed in one study; decreased Glx ratio was observed in SCI subjects with a high NP intensity, suggesting decreased glutamatergic activity that may contribute to their affective distress (62). In the same study, SCI subjects with a lower NP intensity did not show changes in ACC metabolite concentrations compared to healthy controls, suggesting that NP intensity may be reflected in this affective-motivational pain-related region (62).

### Trauma-Induced Brain Changes Following SCI

Based on the articles included here, there is small evidence of volumetric changes in acute and chronic SCI without the presence of NP, but limited evidence on microstructural and metabolite changes in this condition (Table 5).

In acute and incomplete SCI subjects, Chen et al., observed decreased GMV and WMV of regions involved with spatial cognition, memory function, and visual processing (53, 54), which is additionally highlighted by decreases in amplitudes of low-frequency fluctuations within the orbitofrontal cortex and rsFC of the medial visual network (54). These observations could highlight the initial impairment of SCI upon the subjects' movement ability and related brain changes in visuospatial cognitive processing (53, 54). Interestingly, in chronic SCI subjects, increased GMV in areas processing vision (cuneus), sensory information (S1) (56), and affective-motivational/emotional cognition (ACC) was observed (55), whereas decreases in WMV were observed in pathways of projection fibers associated with motor function, i.e., corticospinal tract (56). These volumetric alterations may reflect the damage and cell atrophy to the sensorimotor system, alongside compensatory mechanisms that could accompany chronic SCI subjects without NP, i.e., visual compensation due to lack of sensory feedback (56) and adaptive mechanisms of sensory-pain-related regions in the absence of NP (55, 56). Indeed, S1 and ACC are involved with the sensory-discriminative (85) and affective-motivational/descending control aspects of pain (65, 66), respectively. Microstructural changes in white matter were reported in one study by Gustin et al., showing decreased FA of parietal cortex to midbrain fibers, alongside decreases in MD in cortices involved with executive, motor, sensory, and emotional function (58) (Table 5). Within these regions, decreased FA could reflect impaired axonal integrity or axonal degeneration, whereas decreased MD could reflect changes in neuronal cells, i.e., proliferation or sprouting (30, 83, 86) that may accompany chronic sensorimotor complete SCI without NP (58). Two out of three studies investigating metabolite concentrations in chronic SCI subjects without NP show non-significant changes in the ACC or thalamus (61, 62). However, only one study reported decreases in absolute Ins within the thalamus, which could indicate alterations of glial cells following SCI (64) as Ins is considered a glial marker (38).

### Demographics of SCI Subgroups

In general, recruitment of SCI subjects remains difficult; however, controlling the heterogeneity may limit confounds between studies. As summarized in Table 2, current studies include primarily an older cohort of subjects (majority being male) with a chronic SCI. A recent meta-analysis by Burke et al. (1), which included 1,401 SCI subjects, observed a 53% overall prevalence of NP. Further analysis concluded that NP was found to be more prevalent 1 year postinjury, in people aged 50 or older and tetraplegics (1). However, the prevalence of NP with regard to sex and completeness was not presented due to insufficient data and remains unclear (1). Interestingly, in the studies included here, the presence of NP can be observed in SCI subjects with a range of completeness, level of injury, and pain intensity (Table 2). Three studies were able to include a very homogeneous cohort of SCI subjects, i.e., thoracic level of injury and sensorimotor complete, only delineating the subjects based on NP (58, 61, 63). Another included a restricted criterion of injury level (C5 to T5) although liberal with regard to completeness (56). The remaining studies included an array of injury level and completeness alongside NP (55, 57, 60, 62, 64) (Table 2). As the primary aim of these studies were to observe NP changes in SCI subjects, the sum of the SCI cohort may not allow further differentiation.

Overall, it is unclear in the studies included in this review how the heterogeneity of SCI subjects with NP confounds the brain alterations observed (i.e., structure, function, and metabolite changes). This limiting factor could be explored in future studies within a large cohort allowing comparisons for example between different lesion levels of SCI with NP (thoracic vs. cervical SCI subjects with NP).

### Methodological Considerations

Alongside the demographics of SCI subjects within each study, it is important to consider the method implemented, i.e., modality, quantity of cohort, and statistical thresholds. All studies that investigated structural changes with VBM at a whole brain level corrected for multiple comparisons using regions of interests or spheres (i.e., *p* < 0.05 family-wise error or cluster-level correction; Tables 3–5). Statistical thresholds remain controversial based on factors such as sample size, i.e., a lower threshold (i.e., uncorrected levels) may be more suitable due to a small quantity of subjects; however, the number of false-positive results also increase (87). Utilizing regions of interests as a small volume correction also increases the chance of significant results; hence, regions selected should ideally be in accordance with *a priori* hypothesis shown in a previous work (88). Furthermore, as the VBM studies included here reported volumetric changes by modulating the structural images, the gray matter density changes that accompany NP following SCI remain to be reported. Interestingly, the two DTI studies included here (57, 58) utilized different toolboxes for data analysis, i.e., FMRIB Software Library (89) and Statistical Parametric Mapping (90), which may yield differing results due to differing preprocessing steps. These two studies also differ in subjects recruited as Yoon et al., did not include a SCI cohort without NP (57) (Table 2); hence, more DTI studies are needed. Another discrepancy between each study is the spatial resolutions of the method, i.e., MRS can only investigate particular regions of interest compared to whole brain MRI (i.e., sMRI, DTI, and rsfMRI). To date, only the ACC and thalamus have been reported with MRS; thus, other pain-related regions, i.e., insula, remain to be explored in future studies.

Taken together, methodological differences in future studies may be observed based on the cohort recruited for the hypotheses, neuroimaging modality, or parameters alongside the toolbox and statistical threshold used for data analysis. Therefore, this field may benefit from open data sources and data sharing between SCI centers, in particular the detailed demographics of SCI subjects, the technical parameters of the particular scanner utilized, and the available dataset (i.e., structural MRI, rsfMRI data).

### Limitations

This review is constrained by some important limitations. First, for the purpose of this review, we included a strict criterion (clear information regarding pain) and excluded multiple studies (Supplementary Table 2). Therefore, the overall effects of SCI on the brain may be overlooked. Indeed, previous studies have shown longitudinal structural changes within sensorimotor regions and white matter pathways (91, 92) and microstructural and rsFC changes [see reviews and meta-analysis: (44–47)]. Second, we excluded task-based functional results that may reflect the cortical reorganization due to the presence of NP following SCI, and indeed, the direction of functional reorganization and its correlation to pain can be debated after deafferentation, i.e., in phantom-limb and SCI (20, 21, 71, 76, 77). Third, the papers on supraspinal changes included here were broadly discussed within the neuroimaging method; hence, the underlying interpretations require clarification using a multimodal approach (i.e., neuroimaging, neurophysiology, and clinical assessment) or available animal models. In addition, this review did not discuss studies that investigated pain processing exclusively in the spinal cord, and certainly, this structure is involved in descending pain modulation and pain processing (93–96). Finally, this review did not include a meta-analyses due to the small number of studies alongside the differing methodologies used (Tables 1–5). Instead, an overview of the reported alterations in neuroanatomical landmarks (based on the imaging method) and its correlation with possible biological changes in the SCI condition was reported. This review may also overlook studies that utilized surface-based methods compared to voxel-based approaches.

## Conclusion

The aim of this review was to compile the neuroimaging findings that provide evidence of supraspinal alterations after SCI with NP under no task conditions. Overall, there is some evidence suggesting that the magnitude and presence of NP following SCI results in both adaptive and maladaptive structural plasticity of sensorimotor regions, alongside altered metabolism of brain areas involved with descending pain modulation, pain perception (i.e., ACC and PFC), and sensory integration (i.e., thalamus and S1). In SCI subjects with NP, there remains two reproducible findings: decreases in NAA within the thalamus (60, 61, 64) and decreased GMV of the primary somatosensory cortex (55, 56) (Figure 2). However, based on the fact that only a few studies investigated volumetric, microstructural, and glucose metabolic changes in chronic SCI subjects with NP (55–58), the underlying mechanisms that accompany such changes in these cohorts remain to be further elucidated.

Future cross-sectional studies that aim to disentangle NP within the SCI condition may require stricter constraints in subject cohorts with broader characterization of SCI, i.e., integrity of afferent pathways (complete, incomplete) (51), and pain (including NP) as this may confound the results. In addition to standardizing protocols and scanning sequences, an approach to deal with the problem of cohorts heterogeneity might be the advancement of statistical methods to understand the multiple complex interactions of SCI subjects' characteristics and their effects on neuroplasticity. Cumbersome, longitudinal studies will be vital to understand the alterations of supraspinal areas that accompany NP in SCI subjects. Although there are three studies which have investigated SCI subjects longitudinally (91, 97, 98), with one being a training study (97), changes associated with NP still remain unclear. These studies did not subgroup the SCI cohort into a NP/non-NP group (possibly due to low numbers) (91, 98) (Supplementary Table 2). However, one study reported a positive correlation over 6 months with greater NP intensity and increased iron levels (as measured by quantitative MRI) in the right S2, left ACC, and bilateral cerebellum (98), which may further indicates altered pain processing. In contrast, another study was unable to observe any structural changes in the brain (or spinal cord), which were related to NP longitudinally (91). These results remain to be replicated in future longitudinal studies, which may additionally benefit from the utilization of improved pain phenotyping, i.e., pain drawings, questionnaires, etc. (99) alongside tracking the start of pain development. Taken together, such considerations may allow classifications of the clinical, neurophysiological, and neuroimaging subtypes of NP in SCI subjects, provide additional insight to the complex mechanism of NP, and discover sensitive biomarkers that profile pain and optimize treatment.

## Author Contributions

MH, LM, and VH designed the study and read and assessed full-texts for eligibility. VH performed the literature search, removed duplicates and with screened the records with LM and MH. VH wrote the first draft of the review. All authors read and revised drafts of manuscripts and approved the final version.

### Conflict of Interest

The authors declare that the research was conducted in the absence of any commercial or financial relationships that could be construed as a potential conflict of interest.
