# Supplementary material for: Disentangling the Effects of Spinal Cord Injury and Related Neuropathic Pain on Supraspinal Neuroplasticity: A Systematic Review on Neuroimaging
Source: Front Neurol. 2020 Feb 5;10:1413. doi: 10.3389/fneur.2019.01413 (PMC7013003; doi:10.3389/fneur.2019.01413)
Supplement: Supplementary file 1 [file Data_Sheet_1.pdf]

Supplementary Material

Supplementary Table 1. Outline of numbers of articles throughout search procedure

**PubMed and SCOPUS search (MeSH term combinations)**	**Search total**	**Excluded articles**	**Included articles**	**Duplicate articles**	**n =**
“T” OR “Q” OR “P” OR “SCI” AND “VBM”	56	34	20	8	12
“T” OR “Q” OR “P” OR “SCI” AND “VBCT”	6	1	5	5	0
“T” OR “Q” OR “P” OR “SCI” AND “VBM” AND “NP”	12	8	4	4	0
“T” OR “Q” OR “P” OR “SCI” AND “VBCT” AND “NP”	0	0	0	0	0
“T” OR “Q” OR “P” OR “SCI” AND “DTI”	596	571	25	11	14
“T” OR “Q” OR “P” OR “SCI” AND “DTI” AND “NP	18	14	4	4	0
“T” OR “Q” OR “P” OR “SCI” AND “dMRI”	300	294	6	6	0
“T” OR “Q” OR “P” OR “SCI” AND “dMRI” AND “NP	2	2	0	0	0
“T” OR “Q” OR “P” OR “SCI” AND “DTI*”	367	349	18	18	0
“T” OR “Q” OR “P” OR “SCI” AND “DTI*” AND “NP	2	0	2	2	0
“T” OR “Q” OR “P” OR “SCI” AND “rsfMRI”	27	20	7	4	3
“T” OR “Q” OR “P” OR “SCI” AND “rsfMRI” AND “NP”	0	0	0	0	0
“T” OR “Q” OR “P” OR “SCI” AND “rsfMRI*”	18	10	10	7	3
“T” OR “Q” OR “P” OR “SCI” AND “rsfMRI*” AND “NP”	9	4	5	5	0
“T” OR “Q” OR “P” OR “SCI” AND “rsFC”	16	10	6	5	1
“T” OR “Q” OR “P” OR “SCI” AND “rsFC” AND “NP”	0	0	0	0	0
“T” OR “Q” OR “P” OR “SCI” AND “F N”	85	80	5	1	4
“T” OR “Q” OR “P” OR “SCI” AND “F N” AND “NP”	7	3	4	3	1
“T” OR “Q” OR “P” OR “SCI” AND “fMRI”	426	365	61	30	31
“T” OR “Q” OR “P” OR “SCI” AND “fMRI” AND “NP”	43	30	13	13	0
“T” OR “Q” OR “P” OR “SCI” AND “fMRI*”	288	238	50	48	2
“T” OR “Q” OR “P” OR “SCI” AND “fMRI*” AND “NP”	24	14	10	9	1
“T” OR “Q” OR “P” OR “SCI” AND “MRS”	338	325	13	6	7
“T” OR “Q” OR “P” OR “SCI” AND “MRS” AND “NP”	23	11	12	12	0
“T” OR “Q” OR “P” OR “SCI” AND “PET”	431	425	6	3	3
“T” OR “Q” OR “P” OR “SCI” AND “PET” AND “NP”	27	23	4	4	0
n =	3121	2831	290	208	82
Bibliography search					8
Total					**90**

Summary of article hits after searching with medical subject headings (MeSH) term combinations in PubMeD and SCOPUS. Columns summarise quantity of articles following subsequent steps, i.e. implementing inclusion criteria, removing duplicate articles and bibliography search. *Abbreviations:* dMRI – Diffusion Magnetic Resonance Imaging; DTI – Diffusion Tensor Imaging; fMRI – Functional Magnetic Resonance Imaging; F N – Functional Neuroimaging; MRS – Magnetic Resonance Spectroscopy; NP – Neuropathic Pain; P – Paraplegia; PET – Positron Emission Tomography; Q – Quadriplegia; rsFC – Resting-State Functional Connectivity; rsfMRI – Resting-state fMRI; SCI – Spinal Cord Injury; T – Tetraplegia; VBCT – Voxel-Based Cortical Thickness; VBM – Voxel-Based Morphometry. *Legend:* * = search was performed on abbreviated form of term, i.e. DTI for diffusion tensor imaging, fMRI for functional magnetic resonance imaging

Supplementary Table 2. Articles omitted from qualitative analysis due to lack of pain characterisation, having mixed SCI cohorts or other reasons

References	*Total SCI subjects (n)*	*Pain Assessment*	*SCI with pain/NP (n)*	*Another reason for exclusion*
(Guo et al. 2019)	22	-	-	-
(Cunningham et al. 2018)	7	-	-	-
(Karunakaran et al. 2018)	23	-	-	-
(Seif et al. 2018)	24	-	-	-
(Hawasli et al. 2018)	10	-	-	-
(Ziegler et al. 2018)	15	EMSCI pain questionnaire, NRS (0-10)	7	Mixed cohort for majority of results. Quantitative MRI readouts. SCI subjects were not separated into subgroups of NP.
(Chen et al. 2017)	21	VAS (0-10) at MRI acquisition	15	No specific information on NP. Pain ratings of SCI subjects were not correlated with volumetric changes. SCI subjects were not separated into subgroups of pain.
(Kaushal *et al.*, 2017a)	15	-	-	-
(Kaushal *et al.*, 2017b)	15	-	-	-
(Ilvesmaki et al. 2017)	32	-	-	-
(Pan et al. 2017)	18	-	-	-
(Zheng et al. 2017)	15	-	-	-
(Sun et al. 2017)	23	-	-	-
(Oni-Orisan et al. 2016)	11	-	-	-
(Hou et al. 2016)	25	-	-	-
(Min *et al.*, 2015b)	20	-	-	-
(Grabher et al. 2015)	14	EMSCI pain questionnaire, NRS (0-10)	6	Mixed cohort for majority of results. Changes were primarily related to sensory outcome
(Villiger et al. 2015)	9	ISCIP, NRS (0-10)	5	Training study. Pain reduction during training was included as covariate of no interest in analysis. SCI subjects were not separated into subgroups of NP.
(Zhu et al. 2015)	12	-	-	-
(Koskinen et al. 2014)	34	ISCI basic pain data set	13	SCI subjects were not subgrouped into pain and non-pain groups.
(Hou *et al.*, 2014a)	20	-	-	-
(Hou *et al.*, 2014b)	25	-	-	-
(Freund et al. 2013)	13	-	-	-
(Freund *et al.*, 2012a)	9	-	-	-
(Freund *et al.*, 2012b)	9	-	-	-
(Freund et al. 2011)	10	-	-	-
(Henderson et al. 2011)	20	-	-	-
(Lundell et al. 2011)	19	-	-	-
(Wrigley *et al.*, 2009a)	15	-	-	-
(Guleria et al. 2008)	22	-	-	-
(Wei et al. 2008)	15	-	-	-
(Jurkiewicz *et al.*, 2006)	17	-	-	-
(Crawley et al. 2004)	17	-	-	-
(Puri et al. 1998)	6	-	-	-
(Roelcke et al. 1997)	11	-	-	No specific information on NP assessment.

Overview of subjects' pain assessment as reported in excluded articles. Pain assessment mentioned in study is summarised alongside the total number (n) of SCI subjects mentioned to have pain in relation to the total number of SCI subjects. (–) = no information was provided with regards to pain assessment/number of SCI subjects with pain in the study. Additional information summarises any key points related to pain or neuropathic pain discussed or

mentioned in the study. *Abbreviations:* EMSCI – European Multicenter Study about Spinal Cord Injury; IASP – International Association for the Study of Pain; ISCI – International Spinal Cord Injury; ISCIP – International Spinal Cord Injury Pain Classification; MRI – Magnetic Resonance Imaging; NP – Neuropathic Pain; NRS – Numerical Rating Scale; SCI – Spinal Cord Injury; VAS – Visual Analog Scale. Ranges from 0–10 or 0-100 are commonly used to depict “no pain” to “worst pain imaginable”.
